# A Comparison Study of Virtual Reality Versus Mannequin-Based Simulations for Educating Trainees in the Pediatric Cardiac Critical Care Unit

**DOI:** 10.7759/cureus.91914

**Published:** 2025-09-09

**Authors:** Renee C Willett, Bradford H Ralston, Alejandro Martinez-Herrada, Todd Chang, Gregory Yurasek

**Affiliations:** 1 Cardiac Critical Care Medicine, Children's Hospital of Philadelphia, Philadelphia, USA; 2 Cardiology, Children's National Hospital, Washington, DC, USA; 3 Cardiac Intensive Care Unit, Nicklaus Children's Hospital, Miami, USA; 4 Emergency Medicine, Children's Hospital of Los Angeles, Los Angeles, USA; 5 Pediatric Cardiac Critical Care, Johns Hopkins University School of Medicine, Baltimore, USA

**Keywords:** fellow education, medical education, pediatric cardiac icu, pediatric cardiology, virtual reality

## Abstract

Background: Traditional mannequin-based simulation has been previously demonstrated to be a versatile and effective modality for the education of trainees in the pediatric cardiac intensive care unit (CICU). Unfortunately, creating and maintaining high-fidelity mannequin simulations is resource-intensive. Virtual reality (VR) is an emerging alternative to traditional high-fidelity mannequin simulation for medical education. A previous pilot study by these authors delineated the feasibility of VR in the CICU for the diagnosis and management of patients with hemodynamic compromise. This study sought to compare VR to classic mannequin simulations.

Methods: This prospective, single-center study was approved by the Institutional Review Board (IRB) of a quaternary pediatric center and conducted from September to December of 2021. Four common CICU patient scenarios were developed for both VR and mannequins, including supraventricular tachycardia (SVT), postoperative hypotension after a Norwood procedure, pulmonary hypertensive crisis, and apnea with bradycardia. The VR logic was created by the authors, and the programming was completed by SimX (Mountain View, California, USA). Pediatric cardiology and advanced cardiac critical care fellows completed the first two simulations using either VR or mannequins and then crossed over to complete the last two simulations on the other modality. Fellows were assessed on their completion of a critical action checklist for each scenario as well as by an eight-question post-simulation knowledge test. The average number of checklist items completed was calculated for both mannequin and VR-based scenarios, stratified by type of simulation and year of fellowship.

Results: A total of 14 fellows completed the study. Overall, the average number of checklist items completed and post-test score increased with each postgraduate year. When comparing the overall number of checklist items completed for all of the simulations between mannequin and VR, there was no significant difference in the means (*p* = 0.463). On average, fellows completed two to three out of five critical actions, and the average post-test score was 87%. Fellow questionnaires reflected an interest in VR, a lack of previous VR experience, and a general feeling that VR was more immersive than mannequin simulation. The most common complaint by participants was mild nausea.

Conclusion: Using VR, multiple common CICU scenarios were designed to accurately reflect complex physiologic changes in real-time, creating an immersive and highly realistic simulation environment. Trainees performed no differently with VR simulations as compared to high-fidelity mannequin simulations in the pediatric CICU. Further investigation is required to demonstrate how VR compares to traditional simulation modalities for long-term knowledge retention.

## Introduction

The pediatric cardiac intensive care unit (CICU) cares for patients who may become acutely ill, requiring highly specialized knowledge to prevent decompensation, morbidity, and mortality [1]. While such events require prompt evaluation and management, they occur relatively infrequently. Rotating trainees may, therefore, not have significant exposure to or experience with such events when they arise and may not be given autonomy due to the ongoing need for safety and high-quality outcomes for these patients with little physiological reserve [2]. Traditional mannequin-based simulation has been previously demonstrated to be a versatile and effective modality for the education of trainees in the pediatric CICU [3]. Additionally, such simulation has also been shown to improve trainee readiness and comfort in the CICU, while providing a safe and consequence-free learning environment [4]. Unfortunately, creating and maintaining high-fidelity mannequin simulations is resource-intensive, requiring significant time and cost expenditure [5-8]. Virtual reality (VR) is an emerging alternative to traditional high-fidelity mannequin simulation in medical education [9]. VR provides a cost-effective, repeatable, and standardized means of providing hands-on experience of high-risk events in a low-risk, safe learning environment [9,10]. It has been demonstrated to be an effective method for training physicians in a variety of situations, from diagnostic scenarios to procedural techniques [10-14]. The immersive nature of VR has been shown to cause stress physiology changes in participating physicians, which may create an enhanced learning environment for trainees [15]. VR scenarios can be tailored to replicate complex, high-acuity situations that are unique to the pediatric CICU, offering targeted experiential learning that may be otherwise difficult to achieve. VR's ability to simulate dynamic physiology, critical interventions, and real-time data interpretation closely mirrors the multifaceted decision-making required in the CICU. Such benefits make VR an ideal training methodology in the pediatric CICU, where accurate diagnosis and management require a thorough understanding of a complex variety of inputs from physiological monitors to ventilators.

VR has begun to find its way into the general pediatric intensive care unit (ICU) [16], but there have been limited studies evaluating the use of VR in the pediatric CICU. We have previously demonstrated that VR can realistically simulate clinical scenarios in the pediatric CICU [17]. In that study, a small number of self-selected, board-certified attending physicians were the participants. We demonstrated that experienced clinicians with minimal or no prior VR experience were able to effectively engage in common CICU clinical scenarios. Based on this experience, we determined that VR provided adequate fidelity of hemodynamic feedback for experienced clinicians to recognize and manage these simulated scenarios. This current study builds on that foundational knowledge in an effort to compare performance on VR simulations versus more traditional mannequin-based simulations in CICU scenarios. 

This article was previously presented as a meeting abstract at the Pediatric Cardiac Intensive Care Society 18th Annual International Meeting in Miami, USA, in December 2022.

## Materials and methods

This prospective, single-center study was approved by the Institutional Review Board (IRB) of Children’s National Hospital, and pediatric fellows were enrolled and tested from September 16, 2021, to November 17, 2021. Using the immersive pediatric CICU environment that had previously been developed for the pilot study [17], four new scenarios were created with an emphasis on diagnosis and management of common acute events experienced in the CICU over a period of about six months. The first scenario required fellows to recognize an accelerated heart rate as supraventricular tachycardia (SVT) and understand the difference between a stable and unstable patient based on vital signs. After making the diagnosis, participants had to take appropriate therapeutic steps to perform synchronized cardioversion using external pads and a cardiac monitor/defibrillator (Zoll Medical Corporation, Chelmsford, USA). The second scenario dealt with a neonate with hypoplastic left heart syndrome who was experiencing postoperative hemorrhagic shock immediately after a Norwood procedure. Based on vital signs and physical exam, the participant needed to identify potential etiologies of hypotension and enact therapeutic plans. This scenario included a variety of branch points, with potential outcomes ranging from resolution of hypotension to cardiac arrest. Participants were also able to perform CPR as indicated, and simulated return of spontaneous circulation was possible with appropriate therapy. The third scenario required fellows to identify a patient experiencing a pulmonary hypertensive crisis based on input from the vital signs and ventilator and perform appropriate therapeutic interventions, including medication administration and manual bag-mask ventilation. Finally, the fourth scenario presented an infant with apnea and bradycardia, which required the fellow to consider differential diagnoses not directly related to a cardiac cause.

The topics of each of the scenarios were carefully selected to cover a broad number of potential differential diagnoses and to represent high-risk situations in the CICU. Each of the four scenarios had unique, five-item critical action checklists for appropriate therapeutic management of the diagnoses. These checklists were developed and revised via group consensus by the authors (BR, RW, GY) who hold board certifications in pediatrics, pediatric cardiology, and pediatric critical care medicine. The checklists were then further agreed upon as standard of care by pediatric cardiac critical care intensivists at two other tertiary cardiac institutions. 

The four scenarios were initially developed for traditional mannequin simulation the year prior and were part of a separate initiative to decrease the incidence of arrest. They were carefully adapted to the VR setting. To translate the mannequin simulations to VR, three of the authors (BR, RW, GY) wrote and revised the logic until there was agreement on the rules and reasoning processes that govern how the virtual environment behaves, including game mechanics, narrative flow, and user interactions. The logic was converted to a patient simulation algorithm within a VR platform (SimX, Mountain View, California, USA). Initial alpha versions were tested repeatedly to ensure that the logic provided realistic physiology and progression for each scenario, and appropriate revisions were made over multiple weeks with the SimX and physician teams working closely together over several months. Once approved, the cases were transitioned to the production platform and remained stable throughout the entirety of the study. VR scenarios were run on the SimX platform using Oculus Quest head-mounted displays (Oculus from Facebook, San Jose, California, USA).

**Figure 1:**
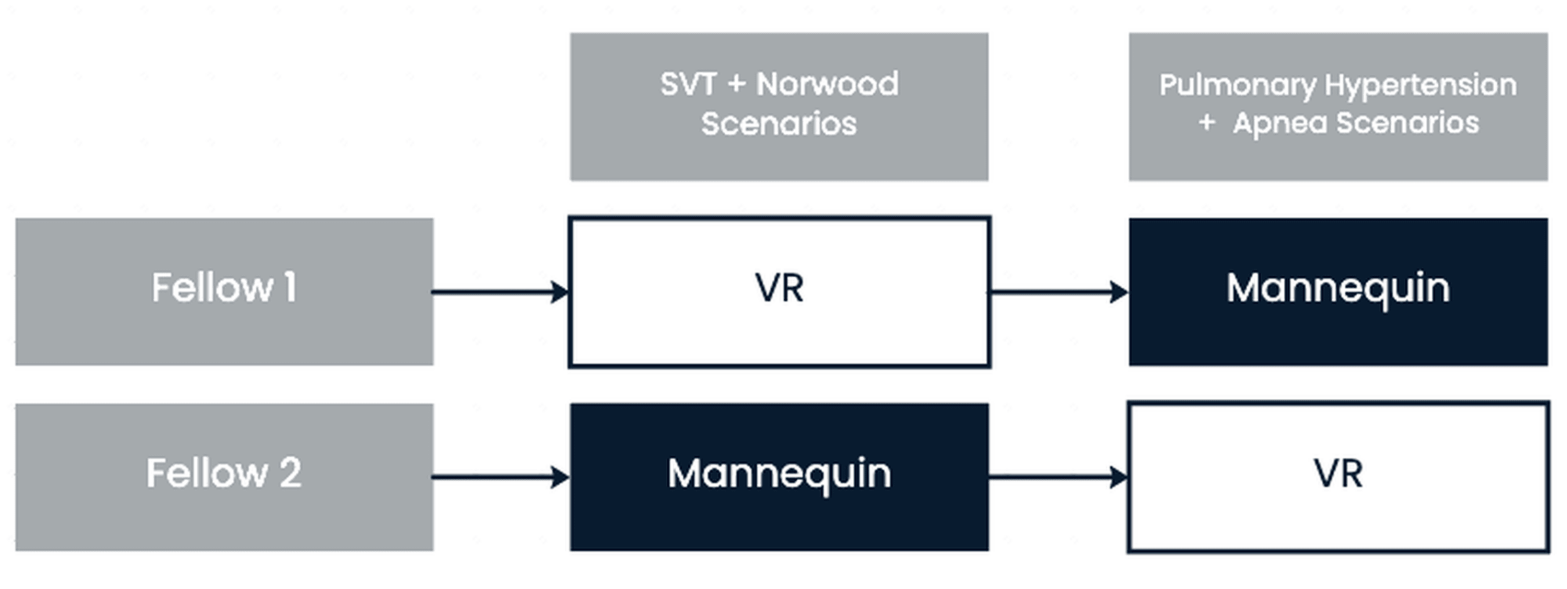
Study flow diagram for two fellows during a single simulation day SVT: Supraventricular tachycardia; VR: Virtual reality

We hypothesized that the VR modality would demonstrate no difference from traditional mannequin simulation regarding the overall participant learning experience. The study, which targeted pediatric cardiology fellows as a convenience sample, was designed as a comparison study with fellows serving as their own controls. Two fellows were tested per simulation day (Figure 1). One fellow would start with VR, and the other would start with mannequin-based simulation. After completing the SVT and Norwood scenarios, fellows would switch simulation modalities and complete the pulmonary hypertensive crisis and apnea/bradycardia scenarios. This design ensured that fellows were evenly split with regard to initial simulation modality.

Authors AM, RW, and GY served as moderators of both the mannequin and VR-based simulations. Prior to starting the VR simulations, all fellows were oriented to the virtual clinical environment as well as to the headset and handset. A standardized orientation to VR, written by the authors, was read to each fellow prior to play, and fellows were given the opportunity to interact in the clinical environment until they felt comfortable with the technology. For the VR simulations, moderators were able to observe the fellow’s actions in real-time via a mirror-streamed display. The fellow played in a first-person view, while the moderator had access to an overhead view. This capability provided the moderator real-time information about the logic and stage progression, as well as the ability to trigger pre-programmed dialogue and a variety of action choices based on participant requests. However, the logic was programmed such that after starting the scenario, no intervention from the moderator was absolutely required. For mannequin simulations, vital sign changes were pre-programmed as per the high-fidelity simulator, and the moderator was able to make changes as needed to accommodate participant actions. Simulations for both modalities were stopped upon completion of the critical action checklist by the fellow or at five minutes, whichever occurred first. Upon conclusion of each simulation in both VR and mannequin modalities, a targeted debrief using the “plus-delta” method was held with the fellow to discuss and reflect on the diagnosis and appropriate management of each scenario. On concluding the two simulations conducted via VR, the fellows were given a questionnaire to reflect on the experience and give feedback. The fellows also completed an eight-question multiple-choice test based on concepts from the four simulations, with two questions for each scenario.

The mean number of checklist items completed was calculated for both mannequin and VR-based scenarios, stratified by simulation as well as fellowship year. The average number of checklist items completed across all four simulations was compared for mannequin versus VR-based simulations using a two-tailed t-test. Free-form results from the VR response survey were summarized.

## Results

A total of 14 fellows completed the study. The fellows were either categorical cardiology fellows or senior fellows in a cardiology subspecialty (Table 1). All enrolled fellows completed all four simulations. All of the fellows completed the simulation post-test as well as questions reflecting on their VR experience. Fellows were asked to grade their prior exposure to VR on a scale of zero to five, with higher scores corresponding to increasing levels of VR experience (Table 1). They were also asked to grade on a scale from zero to five whether they felt that VR enhanced the fidelity of the simulation, made it easier to suspend disbelief, and how useful they felt the modality was to their learning (Table 2). On average, most fellows had positive perceptions of their VR experience.

**Table 1:** Fellows' demographics VR: Virtual reality

Parameter	Frequency	Percentage
Fellows (N=14)		
Year 1	5	35.70%
Year 2	3	21.40%
Year 3	3	21.40%
Year 4	3	21.40%
Gender		
Female	13	92.90%
Male	1	7.10%
Previous VR experience (1-5 Likert)		
1 (No experience)	10	71.40%
2	3	21.40%
3	1	7.10%
4	0	0.00%
5 (Significant experience)	0	0.00%

**Table 2:** Fellows' perceptions of VR VR: Virtual reality

Parameter	Fidelity enhanced	Disbelief	Useful
Average	4.2	4.3	4.9

In general, the average number of checklist items completed increased with each postgraduate year (Table 3). The average post-simulation test score also improved with each postgraduate year (Table 4). On average, the fellows completed two to three of the critical actions for each simulation. Simulations one and three had lower critical action completion scores than simulations two and four. The average post-test score overall for each of the simulations was the same (Table 4).

**Table 3:** Average number of checklist items completed (out of five) stratified by year of fellowship and simulation scenario * indicates statistically significant difference between the average checklist items completed in the mannequin simulation versus the VR simulation VR: Virtual reality

Parameter	Mannequin	VR	P-value
Year of fellowship			
1	1.7	2.2	0.198
2	2.8	2.8	0.5
3	3.3	3.3	0.5
4	2.3	2.5	0.438
Simulation			
Supraventricular tachycardia	1.2	2.6	0.010*
Postoperative hypotension	3.2	2.6	0.77
Pulmonary hypertensive crisis	2.4	2.3	0.517
Hypoxia with bradycardia	2.9	3	0.419
Overall	2.4	2.6	0.463

**Table 4:** Average post-test scores stratified by year of fellowship and simulation scenario VR: Virtual reality

Parameter	Mannequin	VR	Overall
Year of fellowship			
1	80.00%	80.00%	80.00%
2	95.80%	95.80%	95.80%
3	100.00%	100.00%	100.00%
4	76.40%	76.40%	76.40%
Simulation			
Supraventricular tachycardia	85.90%	88.20%	86.90%
Postoperative hypotension	88.20%	85.90%	86.90%
Pulmonary hypertensive crisis	85.90%	88.20%	86.90%
Hypoxia with bradycardia	88.20%	85.90%	86.90%
Overall	87.00%	87.00%	86.90%

The CICU senior fellows also had similar scores between the two modalities, although they performed worse than the third-year fellows. The absolute difference in the number of checklist items completed was most pronounced for simulations one and two. When comparing the overall number of checklist items completed for all the simulations between mannequin and VR, there was no significant difference in the means (*p* = 0.463) (Table 3).

## Discussion

Simulation is a cornerstone of medical education today. Educators need to provide high-quality simulations that reflect the interprofessional nature of the healthcare system while containing costs. The literature has demonstrated that VR can be used effectively in a variety of medical education settings for both technical skills, such as operative technique [18], and nontechnical skills, such as communication, teamwork, and medical knowledge [19,20]. This study builds on our previous work in that we used learners rather than experts to demonstrate that VR can be used successfully in simulating a variety of scenarios in the pediatric CICU for teaching purposes. We also demonstrated that VR was not statistically different as compared to traditional mannequin simulation with regard to the quality of the overall learner experience in these scenarios.

We know from the literature that high-fidelity mannequin simulation is an effective means for teaching trainees at all levels [21,22]. This study was specifically structured to evaluate whether there was a difference in trainee performance and qualitative experience between traditional mannequin and VR simulations. A majority of trainees in this study had at least some experience with high-fidelity mannequin simulations. Thus, this was a comparison of a familiar modality with one that was primarily unfamiliar. The finding that there was no significant difference between the number of checklist items completed by fellows at all levels of training when comparing VR and mannequin suggests that VR is equivalent to a learning tool in this setting. There was a statistically significant difference for the SVT simulation between the mannequin and VR, with learners actually performing better using VR. The pacing box and Zoll cardiac monitor/defibrillator were identical between the mannequin and VR modalities; hence, the etiology of the discrepancy is unclear. We theorize that learners felt safer touching the pacing box in a virtual environment as opposed to the mannequin simulation. Senior fellows also generally performed more poorly than their third-year counterparts in both modalities. Senior fellows included fellows specializing in echocardiography who had not been in the CICU for over six months, thus likely contributing to their worse performance in either modality.

Fellows were able to be adequately oriented to VR in under 10 minutes using a standardized tool. While VR was a new modality for most of the learners in this study, the orientation did not excessively prolong the time needed for fellows to complete the simulation session. Ultimately, we concluded that there was no significant difference between fellow performance in mannequin versus VR simulations. This finding is consistent with a recent systematic review article that suggested there was insufficient existing data to conclusively recommend either VR or traditional simulation as more effective [23].

Our findings have implications for education in the pediatric CICU. With increasing levels of complexity amongst CICU patients as well as an increasing oversight, trainees need easily accessible, standardized learning situations that are high-fidelity and safe. This study demonstrates that highly complex CICU situations, such as the baby struggling immediately after a Norwood surgery, can be modeled with excellent fidelity. Fellows were able to appropriately diagnose and manage these scenarios. Fellows were also able to gain realistic feedback, such as a patient going into cardiac arrest, when they performed incorrect actions for a scenario. Vital sign responses to actions performed were also highly realistic. For example, when fluid was administered, the heart rate decreased while blood pressure increased. With the administration of a vasoactive, both vital signs increased. Since the logic was pre-programmed, the individual running the simulation did not need to trigger the vital sign response or even understand the underlying physiology. Having an intense, highly realistic yet safe learning environment is ideal for teaching trainees how to care for the complex CICU population. Even smaller training programs, which may not have the ability to create and maintain multiple high-fidelity mannequins, may be able to purchase one or two headsets and create or borrow VR scenarios from other training programs. For instance, SimX has a marketplace where centers are able to rent complex modules for training purposes without having to devote the time and money to build scenarios from scratch. 

VR also conveys significant cost savings as compared to a high-fidelity mannequin. The hardware for the VR simulations cost $24,800 for four headsets and all associated technology, such as wireless adapters and controllers. The three-year license with SimX costs $72,000, and each case developed was $1200. This is roughly equivalent to the cost of a new high-fidelity mannequin. However, the VR sets do not degrade at the same rate as the mannequins. The headsets can be used for hundreds of simulations without issue, whereas the life of a mannequin is significantly more limited. Additionally, the mannequin has relatively limited portability. The VR headsets are lightweight, easy to set up and take down, and can be quickly moved from one space to another. The costs and operational characteristics of VR versus a high-fidelity mannequin are summarized in Table 5. 

**Table 5:** Comparison of VR versus high-fidelity mannequin costs and characteristics VR: Virtual reality The table was created by the authors.

Factor	VR	High-fidelity mannequin [5,24]
Initial hardware cost	~$24,800 for 4 headsets + accessories	~$50,000–$200,000 per mannequin
Software/license cost	$72,000 for a 3-year SimX license	May be included or purchased separately
Scenario development cost	$1,200 per case	Varies (often included or manual)
Lifespan/durability	Hundreds of uses; minimal wear	Degrades over time with regular use
Portability	Lightweight; easily transported	Heavy; limited portability
Setup time	Minutes	Can be extensive

There is significant potential for further studies. Here, we have demonstrated that fellows perform similarly when comparing VR and high-fidelity mannequins for this series of simulations. Further investigation is needed to determine whether there are situations in which VR is actually a better educational tool for nontechnical and technical pediatric CICU scenarios. Future studies should also evaluate the feasibility and efficacy of multiplayer CICU scenarios, including multidisciplinary settings. Additionally, future simulations could involve fellows, advanced practitioners, and nurses. VR has the potential to break down geographical barriers by enabling participants in two or more locations to engage in the same simulation, exposing learners to diverse management styles and techniques. Moreover, using VR may be a boon for trainees at smaller centers who might not experience the breadth of complex patients characteristic of larger institutions.

Limitations

This study was a small, single-center study. The generalizability of the study is potentially limited due to the small sample size. The fellows only completed two simulations using VR. Frequently, after the first VR simulation, fellows still felt somewhat uncomfortable with the novel modality. After the second simulation, several mentioned feeling more comfortable with VR and less overwhelmed. If there had been more scenarios, it is possible that with growing comfort, the fellows might have performed even better in VR. Additionally, the five-point checklist may have been insufficient to detect meaningful performance changes. The presence of faculty facilitators during the simulations may also have influenced performance through implicit guidance or support, potentially confounding the effects of the simulation modality. Moreover, the small sample size and single-institution context limit the external validity and may not reflect broader trainee populations. Additionally, participant survey responses may have been subject to bias, including over- or under-reporting, which could have affected the validity of the self-reported data.

While VR simulation for the purpose of training technical medical skills exists, the VR used in this study was inadequate for tasks that emphasize muscle memory, such as intubation or using a pacemaker box. The scenarios were designed to rely on medical knowledge rather than physical tasks. While some of the scenarios involved physical skills such as bagging a patient rapidly, the quality of the bagging technique was not evaluated, as it was not the focus of the simulation. 

## Conclusions

VR holds significant promise as a learning modality for trainees in the CICU. This study demonstrated that learners perform comparably in select pediatric CICU simulations using high-fidelity mannequins and VR. Fellows, on the whole, enjoyed their experience with VR and, on average, agreed that the modality helped them suspend disbelief and enhanced the fidelity of the simulation. VR conveys potential fiscal and portability benefits compared to mannequin simulations. Future studies are needed to determine how VR may be best used to teach healthcare workers in the CICU complex decision-making skills.
